# Clinical Value of MLPA for Prognostic Assessment of Chromosomal Rearrangements and DNA Methylation in Uveal Melanoma

**DOI:** 10.1167/iovs.66.3.51

**Published:** 2025-03-25

**Authors:** Andrea Soltysova, Dana Dvorska, Andrej Ficek, Martina Pecimonova, Marek Samec, Ivana Kasubova, Viera Horvathova Kajabova, Lucia Demkova, Pavel Babal, Jela Valaskova, Zuzana Dankova, Bozena Smolkova, Alena Furdova

**Affiliations:** 1 Department of Molecular Biology, Faculty of Natural Sciences, Comenius University in Bratislava, Bratislava, Slovakia; 2 Institute for Clinical and Translational Research, Biomedical Research Center, Slovak Academy of Sciences, Bratislava, Slovakia; 3 Biomedical Centre Martin, Jessenius Faculty of Medicine in Martin, Comenius University in Bratislava, Martin, Slovakia; 4 Department of Medical Biology, Jessenius Faculty of Medicine in Martin, Comenius University in Bratislava, Martin, Slovakia; 5 Department of Molecular Oncology, Cancer Research Institute, Biomedical Research Center of the Slovak Academy of Sciences, Bratislava, Slovakia; 6 Institute of Pathological Anatomy, Faculty of Medicine, Comenius University in Bratislava, Bratislava, Slovakia; 7 Department of Ophthalmology, Faculty of Medicine, Comenius University in Bratislava, Bratislava, Slovakia; 8 Biobank for Cancer and Rare Diseases, Jessenius Faculty of Medicine in Martin, Comenius University in Bratislava, Martin, Slovakia

**Keywords:** uveal melanoma, MLPA, monosomy 3, tumor suppressor gene, DNA methylation

## Abstract

**Purpose:**

Uveal melanoma (UM) is the most prevalent primary intraocular malignancy in adults, with prognosis significantly influenced by genetic and epigenetic factors. Reliable and cost-effective methods to detect chromosomal aberrations and DNA methylation changes are essential for improving prognostication and informing treatment strategies in UM. This study evaluated the effectiveness of multiplex ligation-dependent probe amplification (MLPA) in detecting UM-specific copy number variations (CNVs) and promoter methylation changes across 25 tumor suppressor genes (TSGs).

**Methods:**

DNA from 58 UM tissues was analyzed with the SALSA MLPA Probemix P027 Uveal melanoma kit, and a subset of 18 samples was further assessed using the SALSA MLPA Probemix ME002-C1 Tumour suppressor mix 2 kit to identify key CNVs and methylation alterations linked to poor prognosis. Validation was carried out with a high-resolution comparative genomic hybridization (CGH) array on 10 samples and the Illumina Infinium Methylation EPIC v1.0 BeadChip array on 25 samples.

**Results:**

Our findings indicate that MLPA is a versatile and robust method for detecting CNVs, showing strong correlations with CGH data and highlighting specific CNV patterns linked to clinical outcomes in UM. However, the ME002-C1 kit showed limited utility for comprehensive methylation analysis, as differential methylation was not observed in the studied TSG loci.

**Conclusions:**

Although MLPA effectively identifies CNVs relevant to UM prognosis, integrating additional methylation-specific approaches could broaden the scope of DNA methylation analysis, offering a more comprehensive molecular understanding of UM that may enhance prognostication and personalized treatment.

Uveal melanoma (UM) is an intraocular malignancy characterized by high metastasis rates and a poor prognosis upon metastatic spread.^1^ Patient outcomes remain limited primarily due to the aggressive metastatic behavior and complex genetic profile of UM. Metastatic potential is strongly correlated with UM-specific chromosomal aberrations, highlighting their importance in prognostic assessment.^2^^,^^3^

Key chromosomal abnormalities frequently observed in UM include the loss of chromosome 3 (3–), gains of chromosome 8 short arm (8q+), and alterations of the long arm of chromosome 1 (1p), as well as both arms of chromosome 6 (6p/q).^4^ Monosomy 3 (M3), the loss of one copy of chromosome 3, and 8p loss (8p–) were found to be independent predictors of poor prognosis.^5^ Studies have shown that M3 is present in approximately 50% of UM cases, with these patients typically experiencing shorter overall survival (OS) compared to those with disomy of chromosome 3 (D3).^6^^,^^7^ Often occurring in conjunction with M3, 8q+ further worsens prognosis, as this aberration is associated with increased tumor proliferation and metastatic capacity. Amplification of 8q often includes genes that drive cell survival and proliferation, exacerbating the aggressiveness and resistance to therapy of UM.^8^ Additionally, alterations in chromosome 1p, specifically the loss of 1p36, are frequently observed in UM, and, in combination with M3, they have been linked to decreased disease-free survival.^9^ Changes in chromosome 6p/q, particularly 6p+ and 6q–, have been reported to possess both favorable and unfavorable prognostic implications. For instance, 6p+ may confer a protective effect, while 6q– is often correlated with aggressive tumor behavior.^1^^,^^10^ These complex patterns of chromosomal aberrations underscore the heterogeneity of UM and its variable clinical course. In addition to these genetic abnormalities, extensive DNA methylation reprogramming has been reported in poor prognosis UMs. Robertson et al.^11^ were the first to demonstrate that BAP1 loss follows M3 and correlates with a distinct global DNA methylation state, suggesting that BAP1 aberrancy drives a metastasis-prone epigenetic profile in M3 UMs. Although M3/BAP1-aberrant tumors shared a uniform DNA methylation pattern, they exhibited genomic and transcriptional heterogeneity, influencing clinical outcomes. In contrast, D3 UMs displayed diverse DNA methylation profiles, reflecting their mutational background. Tumors with *EIF1AX* mutations showed distinct methylation patterns compared to those harboring *SF3B1* or *SRSF2* mutations, providing insight into the biology of these low versus intermediate risk subtypes.^12^ Furthermore, several tumor suppressor genes (TSGs), such as *RASSF1A*, *CDKN2A*, *RNF43*, *GSTP1*, *MEGF10*, *KLF10*, *PRAME*, and *BAP1* exhibit aberrant DNA methylation patterns in UM.^7^^,^^13^^–^^17^ This epigenetic alteration can impair the ability of these genes to regulate crucial cellular processes, including cell cycle control, apoptosis, and genomic stability, thereby contributing to tumor development and progression. Consequently, DNA methylation has emerged as a significant mechanism in UM molecular pathology, offering promising avenues for prognostic biomarkers and targeted therapies.^7^^,^^17^^,^^18^

The identification of chromosomal abnormalities and epigenetic changes provides valuable insights for prognostic evaluation, enabling clinicians to stratify patients based on their risk of metastasis and, despite the limited treatment options, tailor surveillance and treatment strategies accordingly. Genetic alterations were initially explored using a variety of techniques, including standard karyotypic analysis, fluorescence in situ hybridization (FISH), microsatellite analysis, multiplex ligation-dependent probe amplification (MLPA), and single nucleotide polymorphism (SNP) analysis as reviewed by Coupland et al.^3^ Among them, MLPA has emerged as a robust, sensitive, and cost-effective technique for detecting copy number variations (CNVs) and other chromosomal alterations.^19^^,^^20^ The utility of MLPA in UM lies in its ability to simultaneously analyze multiple genetic loci in a single assay, offering a comprehensive profile of key prognostically significant chromosomal abnormalities. Importantly, it enables the detection of both CNVs and DNA methylation changes within a single reaction, making it a versatile tool for exploring genetic and epigenetic alterations.^21^^,^^22^ This dual capability enhances the depth of molecular analysis in UM, supporting the application of MLPA in clinical diagnostics and patient stratification.

This study investigated the versatility of MLPA for comprehensive prognostic profiling and gene-level analysis, focusing on its effectiveness in detecting critical chromosomal aberrations and its adaptability for analyzing TSG DNA methylation in UM. We hypothesized that assessing methylation changes through methylation-specific MLPA (MS-MLPA) could provide additional insights, potentially revealing epigenetic modifications in TSG regions that are pertinent to the progression of UM.

## Methods

### Patient Samples

This study included 58 UM patients who underwent enucleation at the Department of Ophthalmology, Faculty of Medicine, Comenius University in Bratislava, between August 2018 and January 2022 and have been in-depth characterized elsewhere.^7^ Seven of these patients had previously received stereotactic radiosurgery before enucleation. The median age of participants was 66 years, ranging from 32 to 87 years. The distribution of affected eyes was nearly equal, with 51.7% (*n* = 30) in the right eye and 48.3% (*n* = 28) in the left eye; 58.6% of the patients were male (*n* = 34). All excised UM tissues underwent routine histological examination at the Department of Pathology, Faculty of Medicine, Comenius University in Bratislava. Choroidal melanoma (C69.3) was diagnosed in 82.8% of the cases (*n* = 48), and ciliary body melanoma (C69.4) accounted for 17.2% of the cases (*n* = 10). Among the patients, 10 were diagnosed with stage IV disease, and one developed metastasis after primary UM treatment. The study was approved by the ethics committee of the Ruzinov Hospital Bratislava (EK/250/2018). All participants provided written informed consent.

### DNA Extraction and Quality Control

The Gentra Puregene Tissue Kit (Qiagen, Hilden, Germany) was used for DNA extraction from snap-frozen tumor tissues following the manufacturer’s instructions. DNA quantity and purity were evaluated by a NanoDrop 1000 Spectrophotometer (NanoDrop, Wilmington, DE, USA).

### Multiplex Ligation-Dependent Probe Amplification

One hundred nanograms of DNA was used to detect chromosomal rearrangements using the SALSA MLPA Probemix P027 Uveal melanoma kit (MRC Holland, Amsterdam, the Netherlands). This kit includes probes targeting loci on chromosomes 1, 3, 6, and 8 (with seven probes for 1p, 19 for chromosome 3, six for chromosome 6, and six for chromosome 8), as well as 12 control probes. MLPA reactions were conducted according to the manufacturer's instructions. Following amplification, the MLPA products were separated via capillary electrophoresis (3130xl Genetic Analyzer; Applied Biosystems, Waltham, MA, USA). Data analysis was performed using Coffalyser software (MRC Holland).

### Comparative Genomic Hybridization

High-resolution array comparative genomic hybridization (CGH) was conducted on 10 selected UM tissues using the Agilent Microarray Analysis Platform (Agilent Technologies, Santa Clara, CA, USA). After DNA extraction and quality control, 500 ng of DNA was digested and labeled with the SureTag Complete DNA Labeling Kit (Agilent Technologies). The labeled DNA and reference control DNA were combined, applied to the Agilent SurePrint G3 Human Genome CGH+SNP Microarray (2 × 400K), and hybridized for 40 hours at 67°C. Following hybridization, slides were washed and scanned using an Agilent SureScan Microarray Scanner. Data analysis was performed using CytoGenomics software, utilizing the mosaic analysis method to detect genome-wide CNVs and copy-neutral genetic changes. Genomic positions matching those targeted by the MLPA probes were extracted from the whole genome data and compared with the MLPA results.

### Methylation-Specific MLPA

The SALSA MLPA Probemix ME002-C1 Tumour suppressor mix 2 kit (MRC Holland) was used to assess the DNA methylation status of 18 UM tumor samples. The MS-MLPA principle closely resembles traditional MLPA, with one crucial modification: The target sequences recognized by MS-MLPA probes contain a restriction site for methylation-sensitive endonucleases, such as HhaI or HpaII. These enzymes selectively identify cytosine methylation at CpG sites within their recognition sequences. After digestion with these enzymes, amplification of the probe only occurs if the CpG site is methylated, enabling precise detection of the methylation status. This mix includes 41 (MS-)MLPA probes that provide information on CNVs within the sample, 16 reference probes known to maintain stable CNVs across various cancer types, and 27 MS-MLPA probes, each containing an HhaI recognition site. Additionally, 14 reference probes are included that remain unaffected by HhaI digestion, serving as internal controls. The MS-MLPA method is detailed in the MS-MLPA General Protocol (available at www.mrcholland.com).

Briefly, after DNA denaturation and overnight incubation with the probe mix, samples were divided into two tubes, with one tube incubating with HhaI. In this tube, unmethylated DNA was digested and therefore not amplified by PCR. In contrast, methylated DNA was protected from HhaI digestion, allowing these probes to be ligated and subsequently amplified by PCR. The ratio of probe signals between the tubes with and without HhaI provides an estimate of the methylation status. For digestion, restriction endonuclease HhaI (Promega, Madison, WI, USA) was used, and reaction steps were conducted using a SureCycler 8800 thermal cycler (Agilent Technologies). Fragment analysis was performed on an ABI 3500 genetic analyzer (Thermo Fisher Scientific, Waltham, MA, USA), with data analysis carried out using the Coffalyser software. The final methylation status was determined by calculating the ratio of relative probe peaks between the undigested sample (without HhaI) and the corresponding digested sample (with HhaI) for each probe. The baseline level of DNA methylation was defined as the mean methylation level of the reference samples (D3 samples) plus two standard deviations (mean + 2 SD). For each probe in the test sample, methylation was classified as “increased” if the methylation ratio exceeded this baseline. If the methylation level was less than or equal to the baseline, it was classified as “not increased.”

### EPIC BeadChip Array

The methylation status of 25 UM tissues was assessed using the Infinium Methylation EPIC v1.0 BeadChip array (Illumina, San Diego, CA, USA) as described previously.^7^ This high-throughput platform enabled the measurement of individual methylation levels across 850,000 CpG sites. Analysis was conducted by the epigenomic services at Diagenode (Liège, Belgium). DNA methylation was quantified as β values on a scale from 0 (unmethylated) to 1 (fully methylated). For arrays passing quality control, batch effects were corrected with the ComBat algorithm, and data were normalized using the default pipeline in the R ChAMP package (R Foundation for Statistical Computing, Vienna, Austria).^23^ Differentially methylated CpGs (DMGs) were identified based on a false discovery rate–adjusted *P* < 0.05 threshold, with no minimum Δβ threshold for significance.

### Statistical Analysis

Clinical variables were categorized based on the presence of M3, and differences between categorical variables were assessed using the χ^2^ test or Fisher's exact test. Continuous variables are reported as medians with corresponding minimum and maximum values. The Shapiro–Wilk test was used to assess the normality of continuous variables. The tumor volume (TV) was calculated using the formula proposed by Gass^24^: TV = π/6 × (largest basal diameter × width × prominence). Because age and tumor volume did not meet normality assumptions, the non-parametric Mann–Whitney *U* test was applied to compare M3 and D3 groups. OS was defined as the time from the date of the initial examination to the date of death or last follow-up. OS estimates were generated using the Kaplan–Meier method, and differences between groups created based on UM-specific CNV status were assessed with the log-rank test. Univariate Cox proportional hazards regression analysis was performed to calculate hazard ratios (HRs) for individual clinical variables, including M3 status. To identify independent predictors of OS, a multivariate Cox regression model was constructed, including variables that were significant in the univariate analysis, except for the presence of metastasis. Metastasis was excluded from the model because of its correlation with clinical variables linked to poor prognosis, as its inclusion could introduce multicollinearity. The final adjusted model, determined using the backward Wald selection method, is presented. All tests were two-tailed, with *P* < 0.05 considered statistically significant. Statistical analyses were conducted using SPSS Statistics 23 for Windows (IBM, Chicago, IL, USA).

## Results

### Uveal Melanoma-Specific Chromosomal Rearrangements Correlate With Prognosis

Among 58 enrolled patients, 26 tumors were classified as D3 and 30 as M3 based on MLPA analysis. Sample UM37 exhibited a partial loss of chromosome 3 (3q–6p+8q+), and sample UM56 displayed a loss of chromosome 1 short arm (1p–8q+) without any aberrations on chromosome 3. This sample was assigned to the M3 group based on our previous findings. Additionally, four samples showed no UM-specific aberrations and were assigned as D3. Patients with M3 were significantly younger, and this genetic alteration was notably associated with ciliary body melanoma, as well as epithelioid and mixed cell types (Table 1).

**Table 1.** Clinicopathological Characteristics of the Included Patients Stratified by Chromosome 3 Status

	All, *n* (%)	M3, *n* (%)	D3, *n* (%)	*P*
Gender, *n* (%)				
Male	34 (58.6)	16 (50.0)	18 (69.2)	0.139
Female	24 (41.4)	16 (50.0)	8 (30.8)	
Eye, *n* (%)				
Right	30 (51.7)	14 (43.8)	16 (61.5)	0.178
Left	28 (48.3)	18 (56.3)	10 (38.5)	
Age (y), median (range)	66 (32–87)	62 (32–81)	69 (35–87)	**0.049**
Tumor volume (cm^3^), median (range), *n* (%)	1.3 (0.1–5.6)	1.5 (0.2–5.6)	1.2 (0.1–2.6)	0.161
<1.55	37 (63.8)	18 (56.3)	19 (73.1)	0.185
≥1.55	21 (36.2)	14 (43.8)	7 (26.9)	
Diagnosis, *n* (%)				
C69.3	48 (82.8)	22 (68.8)	26 (100.0)	**0.002**
C69.4	10 (17.2)	10 (31.2)	0 (0.0)	
Cell type, *n* (%)				
Epithelioid	11 (19.0)	8 (25.0)	3 (11.5)	**0.034**
Spindle	27 (46.5)	10 (31.3)	17 (65.4)	
Mixed	20 (34.5)	14 (43.7)	6 (23.1)	
Therapy, *n* (%)				
Enucleation	51 (87.9)	29 (90.6)	22 (84.6)	0.485
Enucleation after radiosurgery	7 (12.1)	3 (9.4)	4 (15.4)	

Significant results are highlighted in bold.

**Figure 1.**
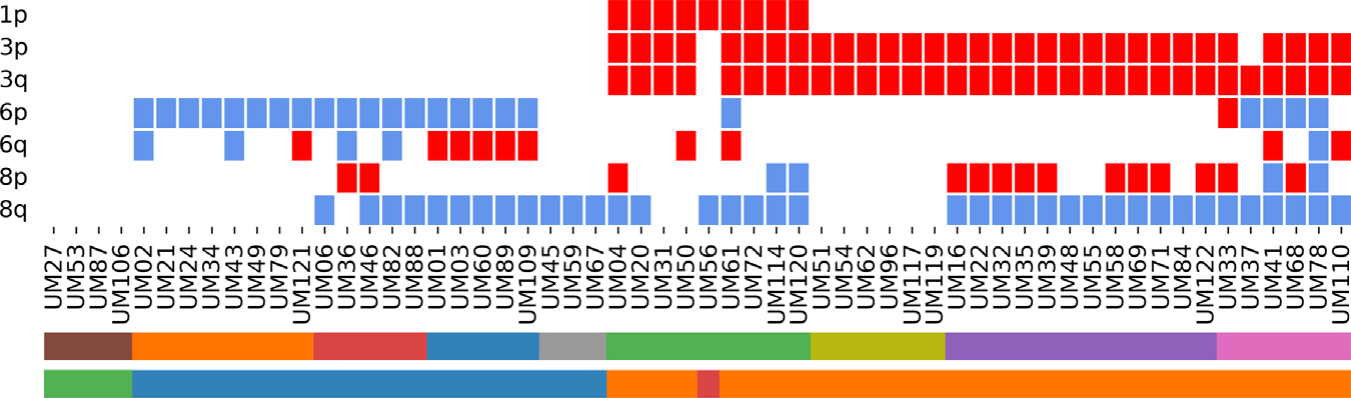
UM-specific chromosomal rearrangements assessed using the SALSA MLPA Probemix P027 Uveal melanoma kit. *Red boxes* indicate chromosomal losses, and *blue boxes* represent gains. For details on the stratification of individual samples, please refer to the legends in Figures 2A and 2B.

Given the established role of chromosomal aberrations in the pathogenesis of UM, particularly the distinct patterns of gains and losses across individual chromosomes, we aimed to analyze their impact on patient survival outcomes. This analysis sought to further elucidate the relationship between chromosomal aberrations and OS, providing insights into their prognostic significance. The patient-specific patterns, as assessed by the P027 Uveal melanoma kit, are presented in Figure 1.

**Figure 2.**
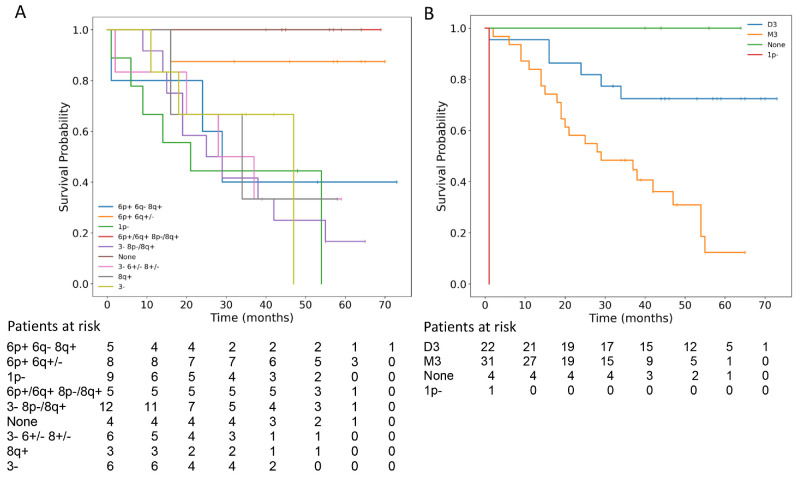
(A, B) Kaplan–Meier analysis of overall survival stratified by combinations of chromosomal aberrations (*P* = 0.014) (**A**) and monosomy 3 (*P* < 0.001) (**B**). D3, disomy of chromosome 3; M3, monosomy of chromosome 3; 1p–, 1p loss without M3.

Losses on chromosomes 1 and 3 were associated with poor survival (Fig. 2A) and showed a strong correlation with 8p– and 8q+ (Fig. 2). Conversely, 6p+ appeared to have a protective effect, even in the presence of abnormalities on chromosome 8, while co-occurrence with 6q– was linked to decreased OS (Fig. 2A). The overall impact of M3 on prognosis is illustrated in Figure 2B and was further confirmed by multivariate analysis (Table 2). Multivariate Cox regression analysis confirmed M3 as an independent risk factor associated with a 3.8-fold increase in mortality risk. This finding underscores the prognostic significance of M3, highlighting its strong association with poorer survival outcomes independent of other clinical factors.

**Table 2.** Cox Regression Analysis for Overall Survival

	Univariate Analysis			Multivariate Analysis		
Risk Factor	HR	95% CI	*P*	HR	95% CI	*P*
Age (continuous)	0.98	0.96–1.01	0.189	—	—	—
Sex	0.64	0.31–1.31	0.219	—	—	—
Affected eye	1.62	0.79–3.36	0.191	—	—	—
Monosomy 3^*^	5.23	2.11–12.97	**<0.001**	3.81	1.40–10.37	**0.009**
Cell type (epithelioid and mixed)	3.81	1.89–7.66	**<0.001**	1.99	0.90–4.41	0.091
Ciliary body melanoma	3.06	1.39–6.72	**0.005**	—	—	—
Tumor volume (continuous)	2.06	1.31–3.24	**0.002**	1.88	1.16–3.04	**0.010**
Ki-67 proliferation ≥ 10%	2.91	1.36–6.23	**0.006**	2.65	1.21–5.79	**0.015**
Metastasis	4.09	1.88–8.88	**<0.001**	NA	—	—

* For this analysis, samples without aberrations were included in the D3 group, and the 1p– UM56 sample was assigned to the M3 group.

Significant results are highlighted in bold.

**Figure 3.**
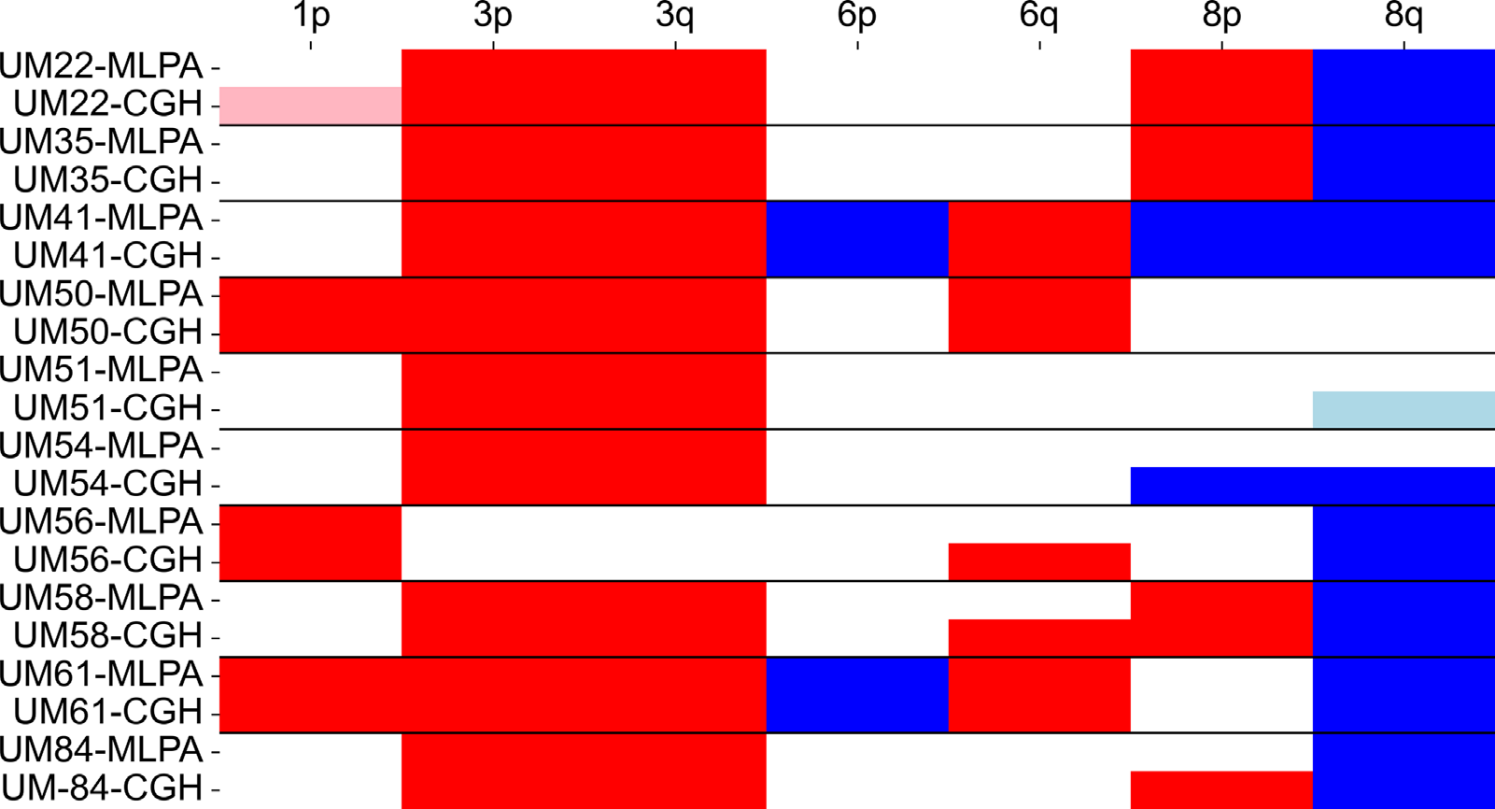
Comparison of findings between MLPA and CGH arrays. *Red* indicates losses, *blue* represents gains, and *lighter colors* highlight partial changes.

To validate the MLPA findings, we compared CNV data with results from the CGH array, which revealed a high degree of concordance between the two methods (Fig. 3). Discordance was observed in seven of the 70 regions analyzed (10%); four cases were associated with losses and three with gains. In all instances, CNVs were missed by MLPA, with four out of the seven discrepancies occurring in the 6q or 8p regions, each covered by two probes.

### Hypermethylation of the Studied TSG Loci Is Infrequent in Tumors With Poor Prognosis

**Figure 4.**
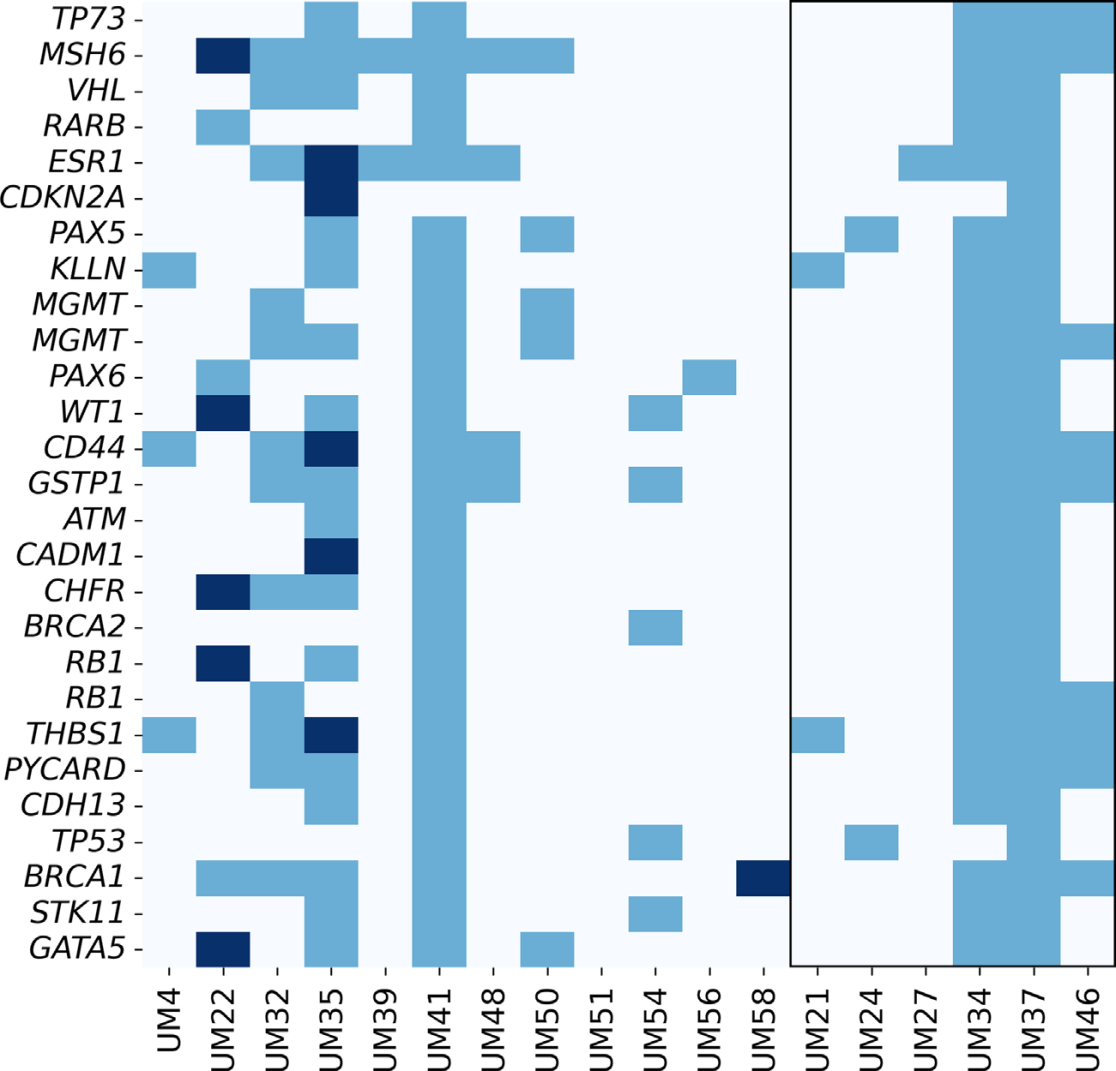
DNA methylation frequency. *Dark blue* indicates increased methylation; *medium blue*, methylation not changed against controls; *light blue*, no signal. Samples highlighted by a *square* are reference samples.

SALSA MLPA Probemix ME002-C1 Tumour suppressor mix 2 provides an option for DNA methylation assessment. Six D3 samples with a favorable prognosis were chosen as the reference to establish baseline methylation levels. Compared to baseline values, only a few probes showed increased methylation in M3 patients (*n* = 12). Overall, we observed relatively infrequent hypermethylation across the analyzed samples, alongside considerable interindividual variability (Fig. 4). The highest frequencies of increased methylation were noted in samples UM22 and UM35. In UM22, genes with elevated methylation included *MSH6*, *WT1*, *CHFR, RB1*, and *GATA5*, whereas UM35 showed increased methylation in *ESR1*, *CDKN2A*, *CD44*, *CADM1*, and *THBS1.* Additionally, patient UM58 exhibited elevated DNA methylation in the *BRCA1* gene (Fig. 4). However, DNA methylation in studied CpGs of selected genes did not differ between the M3 and D3 groups.

**Figure 5.**
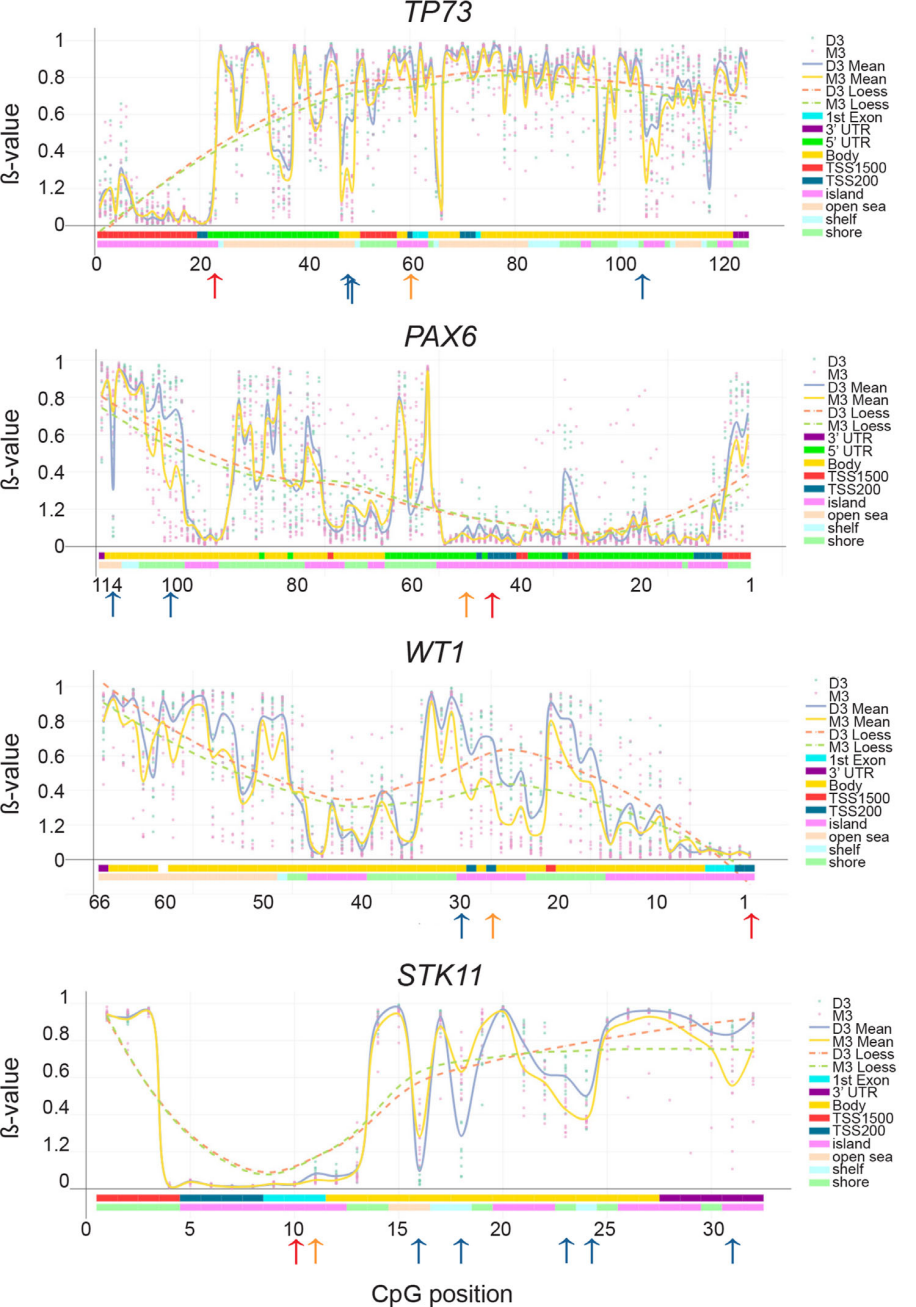
DNA methylation β values for individual probes across the *TP73*, *PAX6*, *WT1*, and *STK11* genes. The *yellow line* indicates mean DNA methylation values for M3 samples, and the *gray* line represents values for D3 samples. The *x*-axis reflects the number of probes analyzed across the Illumina EPIC array for each gene. *Blue arrows* indicate significant differentially methylated CpGs, *orange arrows* highlight CpGs located within CpG islands outside of gene bodies, and *red* indicates the positions of CpGs analyzed using the SALSA MLPA Probemix ME002-C1 Tumor suppressor mix 2. LOESS, locally estimated scatterplot smoothing.

To validate these findings, DNA methylation β values for individual TSGs were extracted from the whole-genome methylation data generated from the analysis of 25 UM tumors using the EPIC array. DMGs were identified at specific loci in 15 out of 25 studied genes, including *TP73*, *RARB*, *ESR1*, *PAX5*, *MGMT*, *PAX6*, *WT1*, *CD44*, *GSTP1*, *CADM1*, *CHFR*, *RB1*, *CDH13*, *TP53*, and *STK11*. Among the 453 analyzed CpGs, 23 exhibited significant differences between M3 and D3, with only four located in CpG island regions outside of gene bodies. Specifically, one was found in *TP73* in the TSS200 region, another in *PAX6* in the 5′ untranslated region (UTR), one in *WT1* in the TSS200 region, and one in *STK11* in the first exon (Fig. 5, Supplementary Table S1). The corresponding Δβ values for these CpGs were –0.16, –0.05, –0.28, and –0.03, respectively, suggesting their hypomethylation in M3 compared to D3. The presented data indicate an absence of hypermethylation in the promoter regions of the studied TSGs in UM patients with poor prognosis.

## Discussion

Multiple molecular methods are available for the identification of chromosomal rearrangements in UM, each with distinct benefits and limitations.^25^^,^^26^ Clinically, FISH and PCR-based techniques are the most commonly used due to their cost effectiveness and targeted precision.^25^^,^^27^^,^^28^ Although CGH and SNP arrays provide broader insights, their higher costs limit their routine use in clinical settings.^29^^,^^30^ The selection of the most suitable method is influenced by the clinical context, available resources, and need for detailed genomic information, with an increasing trend toward integrating multiple techniques for a more personalized and accurate prognostic assessment.

The utilization of the SALSA MLPA Probemix P027 Uveal melanoma kit offers significant advantages, particularly for its high prognostic value, as demonstrated in numerous studies.^19^^,^^20^^,^^30^^,^^31^ In our analysis, we observed a high correlation between MLPA and CGH findings, with a 10% discordance rate. Similarly, Beasley et al.^31^ reported an 8.8% discordance between results from the P027 kit and low-pass whole-genome sequencing. The authors hypothesized that these discordances arise from differences in the sensitivity of each technique, with DNA yield and quality being additional factors. Notably, low-pass whole-genome sequencing also offers the advantage of lower DNA input requirements, making this technique more suitable for limited tumor biopsy samples. On the other hand, CGH provides a comprehensive, high-resolution view of the entire genome, offering the advantage of detecting finer changes such as microdeletions and microduplications; however, its higher cost and the need for sophisticated equipment can limit its routine clinical use. Both methods require high-quality DNA, making them less effective for analyzing degraded or mixed samples, such as necrotic tumor tissues or formalin-fixed, paraffin-embedded samples. In contrast, the targeted approach of MLPA enables precise analysis of UM-specific genes or chromosomal regions, which is advantageous in the case of UM, where a focused gene panel is sufficient for accurate prognosis estimation.

In addition to examining chromosomal rearrangements, several other methodologies have been adopted to accurately identify patients at risk of developing metastatic disease. The 15-gene expression panel (15-GEP), developed by Castle Biosciences (Friendswood, TX, USA), classifies UM patients as Class 1 (low metastatic risk) and Class 2 (high metastatic risk) and is becoming the standard for genetic prognostication of UM patients, primarily in the United States and Canada.^32^^,^^33^ The 15-GEP was prospectively validated by the Collaborative Ocular Oncology Group Report Number 1.^34^ The same group recently validated the superior prognostic accuracy of the integrated 15-GEP/preferentially expressed antigen in melanoma (PRAME) RNA expression classifier compared to 15-GEP alone and clinical prognostic variables.^35^ In Europe, the Liverpool Uveal Melanoma Prognosticator Online (LUMPO) tool was developed to estimate the personalized survival probability of choroidal melanoma patients. It integrates multiple prognostic factors, including clinical, histologic, and genetic data, to improve the accuracy of outcome prediction and estimate survival time and metastatic risk.^36^ The predictive performance of the revised LUMPO version, LUMPO3, which incorporates both chromosome 3 and 8q data, was confirmed in a collaborative multicenter study involving data from seven international ocular oncology centers.^37^ In parallel, the web-based tool Prediction of Risk of Metastasis in Uveal Melanoma (PRiMeUM) was designed using a multivariate approach to estimate the risk of metastasis within 48 months of primary tumor treatment.^38^ However, apart from LUMPO3, no other validated multifaceted tools currently integrate clinical characteristics, histopathologic features, and genetic data for patient prognosis prediction.

Promoter hypermethylation plays a crucial role in regulating TSG expression, often resulting in gene silencing that disrupts cellular control mechanisms and promotes tumorigenesis. In UM, specific DNA methylation patterns correlate with clinical outcomes, and distinct methylation signatures are linked to high-risk subtypes,^7^^,^^12^^,^^16^^,^^18^^,^^39^ thus highlighting the significance of methylation analysis for prognostication and the development of targeted therapies. A reliable and more affordable alternative to whole-genome approaches could greatly expand the accessibility of DNA methylation analysis, making it feasible for wider use in both research and clinical settings. The SALSA MS-MLPA Probemix ME002-C1 Tumour suppressor mix 2 kit has proven to be a valuable tool for identifying DNA methylation in critical TSG regions across various cancers. Additionally, the mix 1 version of this kit expands the analysis to cover a broader spectrum of TSGs. For example, Murria et al.^40^ analyzed DNA methylation of TSGs in breast cancer using the ME001-C1 kit, demonstrating that highly methylated tumors exhibited a higher proportion of CNVs than the sparsely methylated. The same kit was used by Kang et al.^41^ for analysis of DNA methylation in embryonic stem cells. Out of the 24 TSGs analyzed, only three were found to be methylated. The authors reported high consistency between MS-MLPA and pyrosequencing, although less concordance was observed compared to real-time PCR. Furlan et al.^42^ employed both the ME001-C1 and ME002-C1 kits to analyze pancreatic acinar cell carcinoma (ACCs). This dual-kit approach enabled the simultaneous assessment of the methylation status of 34 TSGs and CNVs in 52 genes. Their findings revealed that ACCs did not exhibit extensive global hypermethylation. Only two genes, *RASSF1* and *APC*, were frequently methylated in these tumors. García Martínez et al.^43^ and Magnani et al.^44^ also utilized both kits for TSG methylation analysis in colorectal cancer and pituitary neuroendocrine tumors (PitNETs), respectively. Although early-onset colorectal tumors exhibited significantly fewer methylated genes compared to controls, distinct methylation patterns were observed across different PitNET subtypes. Baranová et al.^45^ recently identified *CDH13* as the most frequently methylated gene in their breast cancer cohort using the ME002-C1 kit, validating their findings obtained using methylation-specific digital droplet PCR. This approach has also been applied in adrenocortical cancers, showing the promising prognostic utility of four DNA methylation markers (*PAX5*, *GSTP1*, *PYCARD*, *PAX6*).^46^ Moelans et al.^47^ used the ME002-B1 kit to investigate methylation markers in ductal carcinoma in situ (DCIS) and invasive breast cancer. They observed frequent promoter hypermethylation of *BRCA2*, *CDH13*, *MSH6*, *PAX5*, *PAX6*, and *WT1* genes in both DCIS and adjacent invasive ductal adenocarcinoma lesions. The study highlighted MS-MLPA as a cost-effective and time-efficient method for evaluating these methylation markers. Similar results were reported for male breast cancer by Kornegoor et al.,^48^ who found hypermethylation of *MSH6*, *WT1*, *PAX5*, *CDH13*, *GATA5*, and *PAX6* in over 50% of cases. In the study by Garinet et al.,^49^ the proportions of methylated CpGs measured by next-generation sequencing showed a strong correlation with methylation status assessed by MS-MLPA (*r* = 0.83). Despite the higher costs associated with next-generation sequencing, the authors consider incorporating methylation analysis into a single NGS experiment to be a more comprehensive approach compared to other techniques, such as pyrosequencing, MS-MLPA, or methylated DNA immunoprecipitation, providing broader insights into methylation patterns.

To the best of our knowledge, DNA methylation in UM has not been previously evaluated using the MS-MLPA method. As we have demonstrated in prior research, the top DEGs identified by the EPIC array exhibited significantly high levels of differential methylation, with Δβ values exceeding 0.7.^7^ However, the genes included in the MLPA ME002-C1 kit exhibit only minor variations in DNA methylation, suggesting that either they are not involved in the prognosis or their regulation in UM may not be primarily driven by methylation changes. The further development of methylation probes by vendors, informed by published whole-genome methylation data, should enhance the utility and effectiveness of MLPA in analyzing TSG promoter methylation in UM.

## Conclusions

Here, we have confirmed the strong concordance between the SALSA MLPA Probemix P027 Uveal melanoma kit and CGH, demonstrating its potential for providing accurate prognostic assessments in UM. The MS-MLPA technique offers a methodological approach that effectively bridges the gap between single-gene methylation analyses and more resource-intensive methods, such as sequencing or array-based analyses. MS-MLPA provides a comprehensive methylation profile without requiring bisulfite conversion, significant financial investment, extensive processing time, or advanced bioinformatics expertise. This makes MS-MLPA a valuable middle-ground approach that offers deeper insights than single-gene assays while circumventing the complexities of large-scale genomic technologies.

However, the utility of MS-MLPA in UM has several limitations. This approach only allows for the assessment of methylation at specific HhaI sites within CpG islands and alterations at loci covered by the assay probes, excluding CpGs located outside these targeted regions. This limitation restricts its versatility in detecting CpGs situated in other regulatory regions, particularly those on shelves or shores, which may be crucial for regulating gene expression. Although findings from the SALSA MS-MLPA ME002-C1 kit correlate well with results from the Illumina EPIC platform, its applicability in UM is further constrained by the lack of significant methylation changes in the studied TSG loci.

## Supplementary Material

Supplement 1
